# Painkiller administration after endoscopic submucosal dissection surgery: a retrospective real-world study

**DOI:** 10.1080/07853890.2025.2499698

**Published:** 2025-05-10

**Authors:** Wu Shanshan, Wang Shuren, Zhang Zongwang

**Affiliations:** a Department of Anesthesiology, Liaocheng People’s Hospital, Shandong University, Liaocheng, China; b Department of Anesthesiology, Liaocheng People’s Hospital, Liaocheng, China

**Keywords:** Endoscopic submucosal dissection, Postoperative pain, Painkiller administration

## Abstract

**Objectives:**

This study aimed to investigate the frequency of analgesic administration within 48 h after endoscopic submucosal dissection (ESD) or its derivative techniques across different segments of the digestive tract, as well as the timing of the initial analgesic administration.

**Materials and Methods:**

This retrospective observational study was built upon previous research. The primary outcomes assessed were the time to initial analgesic administration postoperatively and the frequency of analgesic use within 48 h after ESD surgery. Additionally, factors influencing painkiller administration in ESD patients were analyzed.

**Results:**

Of the 2162 patients included in the study, 570 (26.36%) required postoperative analgesic medications. Most patients required their initial analgesic within 8 h following ESD, with the highest demand observed within 2 h. Multivariate Cox regression analysis indicated that intraoperative administration of ketorolac reduced the likelihood of analgesic administration within 2 h postoperatively (hazard ratio [HR] = 0.35, 95% confidence interval [CI]: 0.15–0.79, *p* = 0.012). The time to first analgesic administration did not differ significantly among patients with varying surgical sites (*H* = 2.043, *p* = 0.843) or surgical methods (*H* = 8.647, *p* = 0.071). Similarly, no significant difference was observed in the frequency of analgesic use within 48 h across different surgical sites (*H* = 3.817, *p* = 0.576). However, patients who underwent endoscopic submucosal excavation (ESE) surgery exhibited a lower frequency of analgesic use compared to those who underwent endoscopic full-thickness resection (EFR) surgery (*p* = 0.038).

**Conclusions:**

A subset of patients undergoing ESD exhibited a need for analgesics within the initial 48-hour postoperative period. Clinicians should carefully assess patients’ pain needs and implement appropriate pain relief measures to improve postoperative outcomes.

**Trial registration:**

Chinese Clinical Trial Registry; ChiCTR2300072854

## Introduction

Acute postoperative pain (APP) is a critical concern that has long garnered the attention of anaesthesiologists, with perioperative pain management constituting a cornerstone of enhanced recovery after surgery (ERAS). Expert consensus guidelines on perioperative analgesia in the operating room have been established and tailored to various surgical sites and procedures [1–4]. However, analgesic options beyond the operating room setting, particularly for minimally invasive endoscopic digestive surgeries, remain an area of ongoing exploration.

Endoscopic submucosal dissection (ESD), a minimally invasive digestive endoscopic technique, offers several advantages, including histological *en bloc* and complete resection, low recurrence rates, and rapid postoperative recovery. It is widely applied for the treatment of early gastrointestinal neoplasms and premalignant lesions [5]. The continuous advancement and clinical application of endoscopic techniques, alongside the utilization of advanced endoscopy-assisted devices, have facilitated the widespread adoption of ESD derivative technologies such as endoscopic submucosal excavation (ESE), endoscopic full-thickness resection (EFR), and submucosal tunnel endoscopic resection (STER). However, these procedures entail both risks and benefits. Postoperative pain, a common yet non-lethal complication, has increasingly drawn the attention of clinicians. Several observational studies have investigated risk factors influencing pain following oesophageal or gastric ESD [6–9]. Research has shown that the prevalence of moderate to severe pain following gastric ESD can reach 63.5%, particularly during the early postoperative period (within 0–6 h post-surgery) [9–11]. Similarly, the incidence of pain following oesophageal ESD is reported to be as high as 49.7% [6]. In addition to identifying influencing factors, previous studies have explored various strategies for alleviating postoperative pain in patients undergoing oesophageal or gastric ESD. Perioperative administration of dexamethasone, proton pump inhibitors (PPIs), transdermal fentanyl, and local or intravenous lidocaine has been found to effectively reduce the incidence of post-ESD pain [10,12–16]. Recent randomized controlled trials (RCTs) have further demonstrated the efficacy of dexmedetomidine and transcutaneous electrical acupoint stimulation (TEAS) in alleviating postoperative pain following oesophageal and gastric ESD [17,18].

However, merely assessing the presence and intensity of postoperative pain in ESD patients is insufficient. It is essential to gain a deeper understanding of the specific anatomical sites and types of minimally invasive surgeries associated with heightened postoperative pain, as well as the frequency, duration, and factors contributing to effective pain relief. Our previous research established and validated predictive models for post-ESD pain [19]. This study focused on analyzing patients who received postoperative analgesics within the first 48 h post-surgery. The study hypothesized that patients administered analgesics within this timeframe were likely experiencing acute postoperative pain. The primary objective was to investigate whether pain intensity varied across different surgical sites and methods. Additionally, the study aimed to explore differences in the timing and frequency of initial analgesic administration among various surgical sites and methods. Identifying the surgical sites, treatment methods, and other factors associated with severe postoperative pain will enable the implementation of targeted analgesic strategies, ultimately enhancing patient outcomes and experiences.

## Methods

### Patients and study design

This retrospective observational study served as a supplementary analysis to our prior research. Data were collected from January 2015 to April 2022 and meticulously entered into an Excel spreadsheet. The Research Ethics Committee of Liaocheng People’s Hospital approved the analysis of routinely collected data. As the study involved a retrospective analysis without patient intervention and ensured the protection of patients’ private information during data extraction, the Ethics Committee granted an exemption from obtaining informed consent in accordance with the principles outlined in the Declaration of Helsinki. The research was registered with the Chinese Clinical Trial Registry (Registration number: ChiCTR2300072854, https://www.chictr.org.cn) and recorded in the Medical Research Registration and Filing Information System (Record number: MR-37-23-021443, https://www.medicalresearch.org.cn).

Patients scheduled to undergo ESD, ESE, EFR, or STER were included, irrespective of the surgical site. Eligible participants were adults aged 18 years or older with an American Society of Anaesthesiologists (ASA) physical status classification of class I to III. Exclusion criteria were as follows: 1) Regular use of postoperative analgesic pumps; 2) Use of analgesics for non-surgical pain; 3) Administration of analgesics either preoperatively or beyond 48 h post-surgery; 4) Severe postoperative complications (e.g. delayed perforation, bleeding necessitating endoscopic hemostasis, or transfer to the intensive care unit within 24 h post-surgery); 5) Requirement for surgical intervention the day following endoscopic treatment; 6) Incomplete follow-up data; 7) Concurrent performance of other types of surgeries. Patients meeting the eligibility criteria were consecutively enrolled to ensure a comprehensive dataset for analysis.

### Data collection

Patients who underwent ESD and its derivative techniques at the Digestive Endoscopy Centre of Liaocheng People’s Hospital for oesophageal and gastrointestinal diseases were initially identified using the Yidu Cloud big data system. Subsequently, eligible patients were further screened according to the inclusion and exclusion criteria using the new electronic medical record system, medical history information system, DoCare Anaesthesia Clinical Information System V5.0, and United Digital Medical Record System.

The initial screening criteria encompassed patients whose surgical titles on the front page of their medical records mentioned ESD, ESE, EFR, or STER. The search parameters included age, gender, height, weight, previous medical history, smoking and alcohol consumption history, surgical history, specific surgical name, anaesthesia record sheet, endoscopic ultrasound report, endoscopic examination and surgery report, and both long-term and short-term medical order records, along with postoperative adverse reactions.

The following variables were ultimately included for analysis: age, gender, body mass index (BMI), ASA classification, history of hypertension and diabetes mellitus, pre-existing pain conditions (including musculoskeletal pain disorders, peripheral neuropathy, and migraines), smoking or alcohol consumption status, surgical history, type of operation (ESD, ESE, EFR, STER, or combined operation), surgical site (oesophagus, esophagogastric junction [EGJ], stomach, duodenum, colorectum, or multisite), operation duration, depth of infiltration (mucosa, submucosa, and lamina muscularis propria), presence or absence of muscular injury, maximum lesion diameter, duration of anaesthesia, intraoperative analgesic usage, postoperative nausea and vomiting (PONV), postoperative fever, analgesic usage within 48 h post-surgery, and length of postoperative hospital stay. To investigate variations in postoperative pain among different age groups, age was categorized into six subgroups: 18–39 years, 40–49 years, 50–59 years, 60–69 years, 70–79 years, and ≥80 years.

### Outcome measures

The primary endpoints of this study were the time to initial administration of postoperative analgesics and the frequency of analgesic use within 48 h following ESD surgery. Additionally, factors influencing painkiller administration in ESD patients during the first 48 h post-surgery were analyzed.

### Statistical analysis

**Figure 1.**
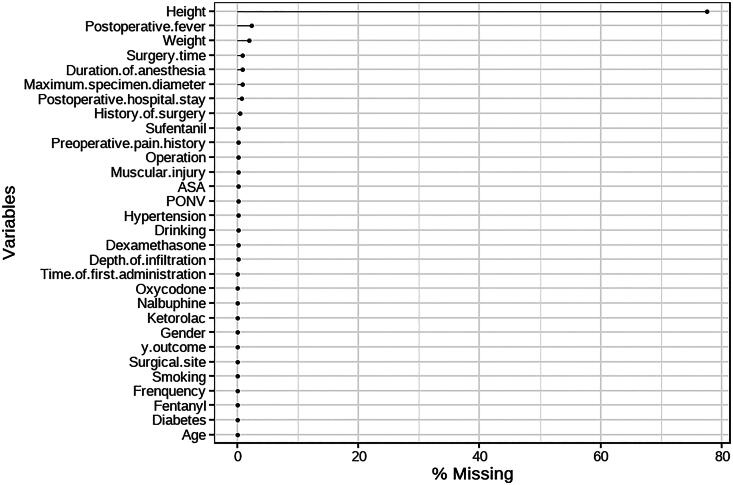
Proportion of missing values for each variable. The proportion of missing data for the height variable is over 70%, whereas the proportion for all other variables is under 5%.

Statistical analyses were conducted using R software (version 4.3.2; http://www.Rproject.org), GraphPad Prism 8.0.2 (GraphPad Software, Inc.), and SPSS software v. 25.0 (IBM Corp., Armonk, New York, USA). As height data were missing in over 70% of the collected patient records, the BMI variable was excluded from the analysis (Figure 1). Missing values for the remaining variables accounted for less than 5% of the dataset and were imputed five times using the multiple imputation method with the mice package in R software. It was assumed that missing data occurred at random, dependent on other observed variables, but independent of the values of the missing variables themselves. No missing data were observed for outcome indicators. All result analyses were based on the multiple imputation datasets selected according to the minimum Akaike’s information criterion (AIC).

The Kolmogorov-Smirnov test was applied to assess the distribution of quantitative variables. Skewed continuous quantitative variables were expressed as median (IQR), while categorical qualitative variables were reported as frequency (n) and percentage (%). Group comparisons of quantitative data were performed using the Wilcoxon Mann-Whitney test, while the Kruskal-Wallis test was applied for comparisons involving more than two groups with non-normally distributed data. Categorical data were compared between groups using the chi-square test or Fisher’s exact test, as appropriate.

Binary logistic regression analysis was employed to identify influencing factors of post-ESD painkiller utilization. Given the presence of missing data for certain exposures, secondary analyses were conducted by repeating the main analyses using the raw dataset with missing values (See Supplementary Table A). A forest plot visualizing the results of the multivariate COX regression analysis was generated with the forest plot package in R software. All statistical tests were two-sided, with *p*-values < 0.05 considered statistically significant.

## Results

**Figure 2.**
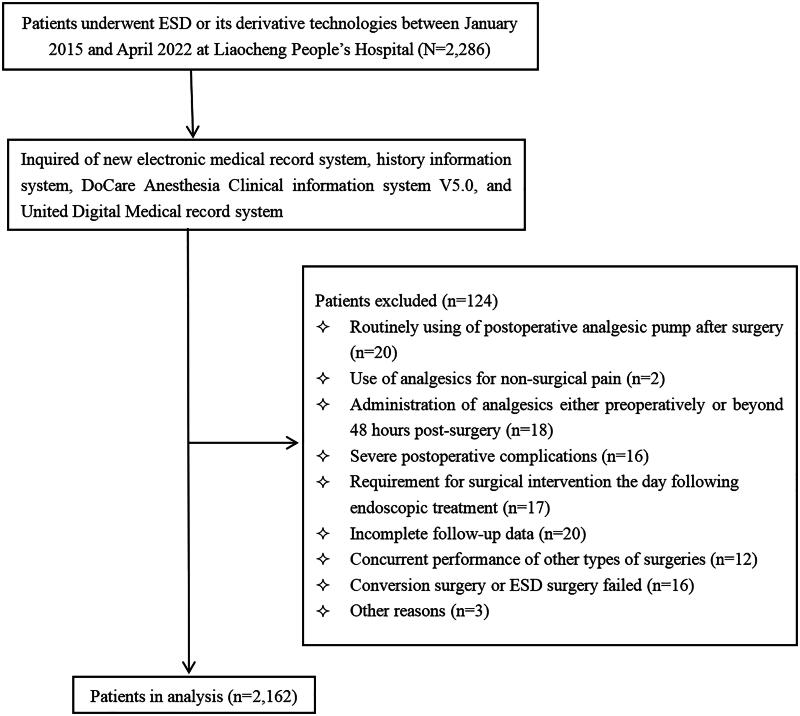
The flowchart of patient selection.

A total of 2,286 patients were screened for eligibility at the Digestive Endoscopy Centre of Liaocheng People’s Hospital. Following exclusions, 2,162 subjects were included in the final analysis. The study flow diagram is presented in Figure 2, while the clinicopathologic characteristics of the patients are detailed in Table 1. Among the included patients, 570 (26.4%), 419 (19.4%), and 267 (12.3%) experienced APP, PONV, and postoperative fever, respectively. The distribution of patients according to the anatomical site included 966 (44.7%) oesophageal, 210 (9.7%) EGJ, 700 (32.4%) stomach, 16 (0.7%) duodenum, and 193 (8.95%) colorectum cases. The incidence of postoperative analgesic administration was 35.9% (347/966) in the oesophagus, 14.8% (31/210) in the EGJ, 19.1% (134/700) in the stomach, 37.5% (6/16) in the duodenum, and 12.4% (24/193) in the colorectum.

**Table 1.** Clinical characteristics and procedure-related index in 2162 patients who underwent ESD or its derivative technologies.

Variables	Total (*n* = 2162)	No pain (*n* = 1592)	Pain (*n* = 570)
Age (y, %)			
18–39	78 (3.6)	59 (3.7)	19 (3.3)
40–49	196 (9.1)	153 (9.6)	43 (7.5)
50–59	631 (29.2)	471 (29.6)	160 (28.1)
60–69	797 (36.9)	592 (37.2)	205 (36.0)
70–79	412 (19.1)	283 (17.8)	129 (22.6)
≥80	48 (2.2)	34 (2.1)	14 (2.5)
Gender (%)			
Female	970 (44.9)	693 (43.5)	277 (48.6)
Male	1192 (55.1)	899 (56.5)	293 (51.4)
Smoking (%)			
Non-smokers	1450 (67.1)	1077 (67.7)	373 (65.4)
Present smokers	573 (26.5)	438 (27.5)	135 (23.7)
Former smokers	139 (6.4)	77 (4.8)	62 (10.9)
Drinking (%)			
Non-drinkers	1411 (65.3)	1019 (64.0)	392 (68.8)
Present drinkers	655 (30.3)	512 (32.2)	143 (25.1)
Former drinkers	96 (4.4)	61 (3.8)	35 (6.1)
ASA (%)			
I	23 (1.1)	21 (1.3)	2 (0.4)
II	2016 (93.2)	1448 (91.0)	568 (99.6)
III	123 (5.7)	123 (7.7)	0 (0.0)
History of surgery (%)			
0	1500 (69.4)	1159 (72.8)	341 (59.8)
1	662 (30.6)	433 (27.2)	229 (40.2)
Hypertension (%)			
0	1663 (76.9)	1309 (82.2)	354 (62.1)
1	499 (23.1)	283 (17.8)	216 (37.9)
Diabetes (%)			
0	1872 (86.6)	1483 (93.2)	389 (68.2)
1	290 (13.4)	109 (6.8)	181 (31.8)
Preoperative pain history (%)			
0	1456 (67.3)	1156 (72.6)	300 (52.6)
1	706 (32.7)	436 (27.4)	270 (47.4)
Surgical.site (%)			
Esophagus	966 (44.7)	619 (38.9)	347 (60.9)
EGJ	210 (9.7)	179 (11.2)	31 (5.4)
Stomach	700 (32.4)	566 (35.6)	134 (23.5)
Duodenum	16 (0.7)	10 (0.6)	6 (1.1)
Colorectum	193 (8.9)	169 (10.6)	24 (4.2)
Multisite	77 (3.6)	49 (3.1)	28 (4.9)
Operation (%)			
ESD	1726 (79.8)	1273 (80.0)	453 (79.5)
ESE	231 (10.7)	184 (11.6)	47 (8.2)
EFR	65 (3.0)	42 (2.6)	23 (4.0)
STER	101 (4.7)	73 (4.6)	28 (4.9)
Combined operation	39 (1.8)	20 (1.3)	19 (3.3)
Maximum specimen diameter (cm, median [IQR])	2.50 [1.50, 4.00]	2.40 [1.17, 3.50]	3.00 [2.00, 4.20]
Depth of infiltration (%)			
Mucous layer	1644 (76.0)	1230 (77.3)	414 (72.6)
Submucosa	119 (5.5)	71 (4.5)	48 (8.4)
Lamina muscularis propria	399 (18.5)	291 (18.3)	108 (18.9)
Muscular injury (%)			
0	1482 (68.5)	1114 (70.0)	368 (64.6)
1	680 (31.5)	478 (30.0)	202 (35.4)
Surgery time (min, median [IQR])	64.00 [46.00, 85.00]	60.00 [45.00, 77.00]	75.00 [52.00, 105.00]
Duration of anesthesia (min, median [IQR])	77.00 [60.00, 99.00]	72.00 [58.00, 89.00]	90.00 [70.00, 120.00]
Fentanyl (%)			
0	136 (6.3)	114 (7.2)	22 (3.9)
1	2026 (93.7)	1478 (92.8)	548 (96.1)
Sufentanil (%)			
0	1976 (91.4)	1465 (92.0)	511 (89.6)
1	186 (8.6)	127 (8.0)	59 (10.4)
Oxycodone (%)			
0	2159 (99.9)	1590 (99.9)	569 (99.8)
1	3 (0.1)	2 (0.1)	1 (0.2)
Nalbuphine (%)			
0	2132 (98.6)	1569 (98.6)	563 (98.8)
1	30 (1.4)	23 (1.4)	7 (1.2)
Ketorolac (%)			
0	2071 (95.8)	1535 (96.4)	536 (94.0)
1	91 (4.2)	57 (3.6)	34 (6.0)
Dexamethasone (%)			
0	2112 (97.7)	1551 (97.4)	561 (98.4)
1	50 (2.3)	41 (2.6)	9 (1.6)
PONV (%)			
0	1743 (80.6)	1355 (85.1)	388 (68.1)
1	419 (19.4)	237 (14.9)	182 (31.9)
Postoperative fever (%)			
0	1895 (87.7)	1427 (89.6)	468 (82.1)
1	267 (12.3)	165 (10.4)	102 (17.9)
Postoperative hospital stay (d, median [IQR])	5.00 [4.00, 6.00]	5.00 [4.00, 6.00]	6.00 [5.00, 7.00]

EGJ: esophagogastric junction; ESD: endoscopic submucosal dissection; ESE: endoscopic submucosal excavation; EFR: endoscopic full-thickness resection; STER: submucosal tunnel endoscopic resection. PONV: postoperative nausea and vomiting.

Regarding surgical procedures, ESD was the most common technique, accounting for 79.8% of cases, followed by ESE (10.7%), EFR (3.0%), and STER (4.7%). The maximum specimen diameter was significantly larger in patients who received postoperative analgesics compared to those without analgesics [3.00 (2.00, 4.20) cm vs. 2.40 (1.17, 3.50) cm]. The median duration of surgery and anaesthesia was 64 min and 77 min, respectively. Patients without postoperative pain had a shorter hospital stay than those with pain [5.00 (4.00, 6.00) vs. 6.00 (5.00, 7.00)].

A total of 2,026 (93.7%) patients received fentanyl for intraoperative analgesia. However, only a small proportion of patients required supplementary analgesics, including oxycodone (3 patients, 0.1%), nalbuphine (30 patients, 1.4%), and ketorolac (91 patients, 4.2%). Additionally, smoking and drinking habits, as well as a history of preoperative pain and previous surgeries, were identified as potential risk factors for exacerbating postoperative pain.

The study conducted a univariate and multivariate analysis to identify risk factors associated with analgesic use post-ESD surgery. In the multivariate analysis, several factors were identified as independent risk factors associated with the increased need for analgesics following ESD surgery, including current smoking (odds ratio [OR] 1.77, 95% confidence interval [CI] 1.18–2.65, *p* = 0.006), former smoking ([OR] 3.51, 95% CI 1.85–6.68, *p* < 0.001), history of previous surgery ([OR] 1.87, 95% CI 1.47–2.38, *p* < 0.001), preoperative pain history ([OR] 2.46, 95% CI 1.94–3.12, *p* < 0.001), hypertension ([OR] 1.64, 95% CI 1.23–2.19, *p* < 0.001), diabetes mellitus ([OR] 4.99, 95% CI 3.56–7.00, *p* < 0.001), larger specimen size ([OR] 1.13, 95% CI 1.06–1.21, *p* < 0.001), lesions located in the submucosa ([OR] 4.82, 95% CI 2.90–7.99, *p* < 0.001) and lamina muscularis propria ([OR] 2.27, 95% CI 1.29–3.99, *p* = 0.004), muscular injury ([OR] 1.65, 95% CI 1.17–2.31, *p* = 0.004), PONV ([OR] 2.76, 95% CI 2.10–3.62, *p* < 0.001), postoperative fever ([OR] 1.64, 95% CI 1.18–2.28, *p* = 0.003), and surgical sites involving the oesophagus and duodenum.

Conversely, current drinkers ([OR] 0.46, 95% CI 0.31–0.69, *p* < 0.001), former drinkers ([OR] 0.43, 95% CI 0.21–0.92, *p* = 0.028), and patients undergoing STER ([OR] 0.35, 95% CI 0.17–0.73, *p* = 0.005) were associated with a lower likelihood of requiring analgesics post-ESD (Table 2).

**Table 2.** Univariate and multivariate analysis of risk factors for post-ESD pain.

Variables	Univariate analysis		Multivariate analysis	
	Odds ratio (95%CI)	*P* value	Odds ratio (95%CI)	*P* value
Age (y)				
(18–39) *vs.* (40–49)	0.87 (0.47–1.62)	0.666		
(18–39) *vs.* (50–59)	1.05 (0.61–1.82)	0.848		
(18–39) *vs.* (60–69)	1.08 (0.63–1.85)	0.792		
(18–39) *vs.* (70–79)	1.42 (0.81–2.47)	0.222		
(18–39) *vs.* (≥80)	1.28 (0.57–2.87)	0.552		
Gender (female *vs.* male)	0.82 (0.67–0.99)	0.037	0.82 (0.61–1.10)	0.179
Smoking				
non-smokers *vs*. present smokers	0.89 (0.71–1.12)	0.312	1.77 (1.18–2.65)	0.006
non-smokers *vs*. Former smokers	2.32 (1.63–3.31)	< 0.001	3.51 (1.85–6.68)	< 0.001
Drinking				
non-drinkers *vs*. present drinkers	0.73 (0.58–0.90)	0.004	0.46 (0.31–0.69)	< 0.001
non-drinkers *vs*. former drinkers	1.49 (0.97–2.30)	0.07	0.43 (0.21–0.92)	0.028
ASA				
I *vs.* II	4.12 (0.96–17.62)	0.056		
I *vs.* III	0.00 (0.00–Inf)	0.966		
History of surgery (no *vs.* yes)	1.80 (1.47–2.20)	< 0.001	1.87 (1.47–2.38)	< 0.001
Hypertension (no *vs.* yes)	2.82 (2.28–3.49)	< 0.001	1.64 (1.23–2.19)	< 0.001
Diabetes (no *vs.* yes)	6.33 (4.87-8.23)	< 0.001	4.99 (3.56–7.00)	< 0.001
Preoperative pain history (no *vs.* yes)	2.39 (1.96–2.91)	< 0.001	2.46 (1.94–3.12)	< 0.001
Surgical site				
Esophagus *vs.* EGJ	0.31 (0.21–0.46)	< 0.001	0.22 (0.14–0.36)	< 0.001
Esophagus *vs.* Stomach	0.42 (0.34–0.53)	< 0.001	0.28 (0.20–0.39)	< 0.001
Esophagus *vs.* Duodenum	1.07 (0.39–2.97)	0.896	0.87 (0.25–3.04)	0.830
Esophagus *vs.* Colorectum	0.25 (0.16–0.40)	0.001	0.19 (0.10–0.37)	< 0.001
Esophagus *vs.* Multisite	1.02 (0.63–1.65)	0.938	0.44 (0.22–0.89)	0.023
Operation				
ESD *vs*. ESE	0.72 (0.51–1.01)	0.054	0.64 (0.37–1.11)	0.110
ESD *vs*. EFR	1.54 (0.92–2.59)	0.104	1.25 (0.57–2.75)	0.580
ESD *vs*. STER	1.08 (0.69–1.69)	0.743	0.35 (0.17–0.73)	0.005
ESD *vs*. Combined operation	2.67 (1.41–5.05)	0.002	1.32 (0.51–3.36)	0.566
Maximum specimen diameter (cm)	1.20 (1.14–1.26)	< 0.001	1.13 (1.06–1.21)	< 0.001
Depth of infiltration				
Mucous layer *vs*. Submucosa	2.01 (1.37–2.95)	< 0.001	4.82 (2.90–7.99)	< 0.001
Mucous layer *vs*. Lamina muscularis propria	1.10 (0.86–1.41)	0.439	2.27 (1.29–3.99)	0.004
Muscular injury (no *vs.* yes)	1.28 (1.04–1.57)	0.017	1.65 (1.17–2.31)	0.004
Surgery time (min)	1.01 (1.01–1.01)	< 0.001	1.00 (1.00–1.01)	0.360
Duration of anesthesia (min)	1.01 (1.01–1.01)	< 0.001	1.00 (1.00–1.01)	0.184
Fentanyl (no vs. yes)	1.92 (1.20–3.06)	0.006	0.79 (0.38–1.63)	0.518
Sufentanil (no vs. yes)	1.33 (0.96–1.84)	0.084		
Oxycodone (no vs. yes)	1.40 (0.13–15.44)	0.785		
Nalbuphine (no vs. yes)	0.85 (0.36–1.99)	0.705		
Ketorolac (no vs. yes)	1.71 (1.10–2.64)	0.016	1.29 (0.76–2.19)	0.349
PONV (no *vs.* yes)	2.68 (2.14–3.35)	< 0.001	2.76 (2.10–3.62)	< 0.001
Postoperative fever (no *vs.* yes)	1.88 (1.44–2.46)	< 0.001	1.64 (1.18–2.28)	0.003

EGJ: esophagogastric junction; ESD: endoscopic submucosal dissection; ESE: endoscopic submucosal excavation; EFR: endoscopic full-thickness resection; STER: submucosal tunnel endoscopic resection; PONV: postoperative nausea and vomiting.

**Figure 3.**
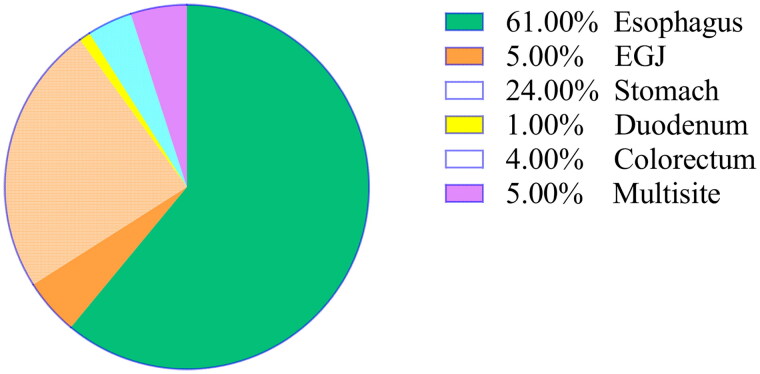
The pie chart illustrates the use of postoperative analgesic drugs in patients with different surgical sites. Among all patients receiving postoperative analgesics, those who underwent oesophageal endoscopic surgery comprised the largest proportion at 61%, followed by gastric endoscopic surgery at 24%. EGJ: esophagogastric junction.

**Figure 4.**
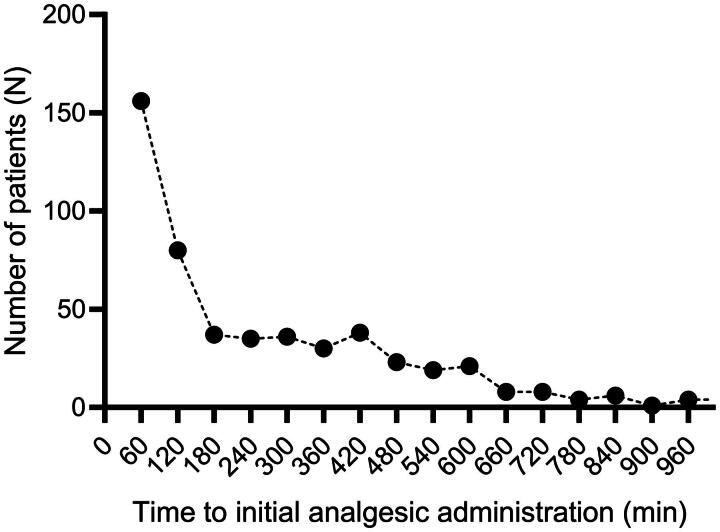
Number of patients receiving analgesics at different time intervals within 48 h postoperatively. A total of 156 patients received their first dose of analgesics within 60 minutes post-surgery. An additional 80 patients received their initial analgesic treatment between 60 and 120 minutes after the procedure. During subsequent time intervals (2–3 hours, 3–4 hours, 4–5 hours, 5–6 hours, and 6–7 hours post-operation), approximately 35 patients per interval initiated analgesic use. Within the 7–8 hour period following surgery, 23 patients were administered analgesics for the first time. After 8 hours, the number of patients requiring further doses of analgesics progressively declined.

Among all patients, 570 (26.36%) received at least one dose of analgesia following ESD. The pie chart (Figure 3) illustrates that oesophageal surgery accounted for the largest proportion (61%) of patients who received postoperative analgesics, followed by stomach surgery at 24%. Figure 4 shows that the majority of patients were administered their initial dose of analgesics within 8 h after ESD, with the highest frequency observed during the first 2 h.

**Figure 5.**
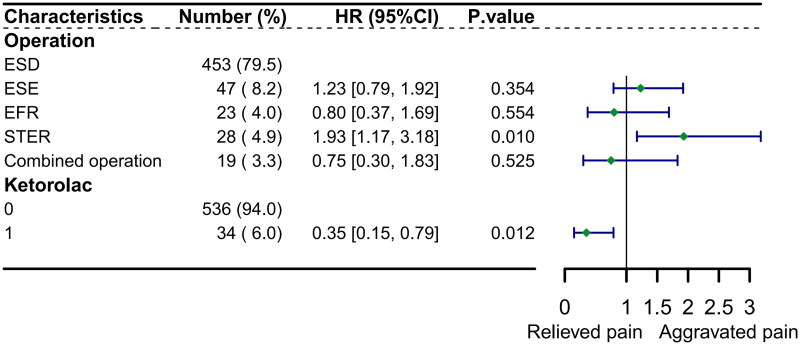
Forest plot of multivariate cox regression analysis for the incidence of initial analgesic use within 2 hours post-surgery. ESD: endoscopic submucosal dissection. ESE: endoscopic submucosal excavation; EFR: endoscopic full-thickness resection; STER: submucosal tunnel endoscopic resection.

Cox regression analysis was performed to identify factors influencing the administration of analgesics within 2 h. Multivariate Cox regression analysis demonstrated that patients who underwent STER were significantly more likely to receive analgesics within 2 h post-surgery (hazard ratio [HR] 1.93, 95% CI 1.17–3.18, *p* = 0.010). Conversely, intraoperative administration of ketorolac was associated with a reduced likelihood of requiring early postoperative analgesia ([HR] 0.35, 95% CI 0.15–0.79, *p* = 0.012). The forest plot depicting these findings is presented in Figure 5.

**Figure 6.**
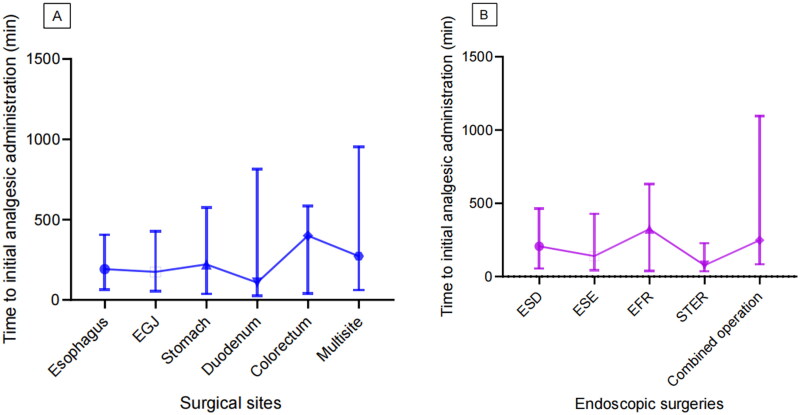
The comparison of time to initial administration of analgesics across different surgical sites (A) and types of surgery (B). EGJ: esophagogastric junction; ESD: endoscopic submucosal dissection; ESE: endoscopic submucosal excavation; EFR: endoscopic full-thickness resection; STER: submucosal tunnel endoscopic resection.

Additionally, a comparative analysis was conducted to investigate whether the timing of the first analgesic administration varied according to surgical sites and procedure types. Statistical analysis revealed no significant differences in the time to initial analgesic administration among patients with different surgical sites (*H* = 2.043, *p* = 0.843, Figure 6A) or different types of surgery (*H* = 8.647, *p* = 0.071, Figure 6B).

**Figure 7.**
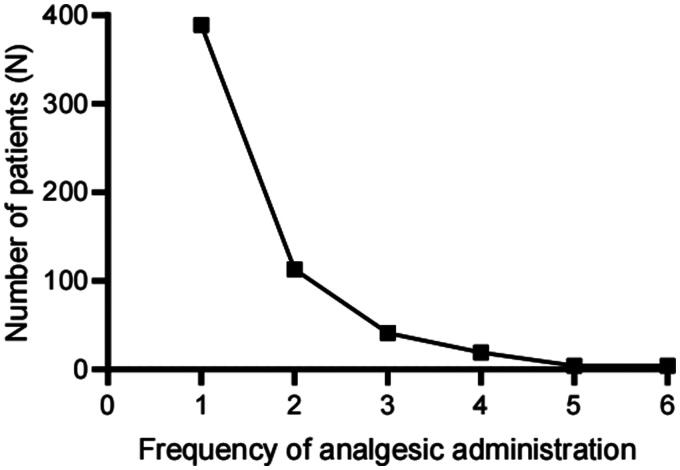
Distribution of patients receiving analgesics at different frequencies within 48 h post-surgery. Of the 570 patients who received analgesic drugs, 389 (68.2%) used the medication once within 48 hours post-surgery. Additionally, 113 patients (19.8%) required two doses of analgesics during this period.

**Figure 8.**
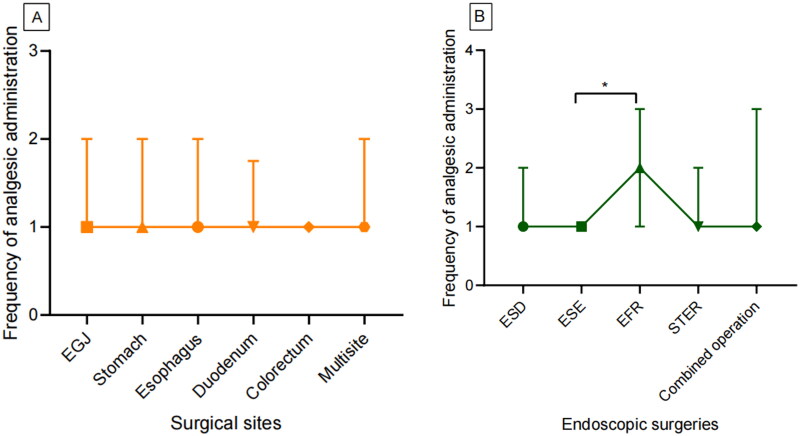
Comparison of postoperative analgesic utilization frequency by surgical procedure (A) and anatomical site (B). A significantly lower frequency of analgesic administration was observed in patients undergoing ESE treatment compared to the EFR cohort. *p < 0.05. EGJ: Esophagogastric junction; ESD: endoscopic submucosal dissection; ESE: endoscopic submucosal excavation; EFR: endoscopic full-thickness resection; STER: submucosal tunnel endoscopic resection.

It was hypothesized that an increased frequency of analgesic administration within 48 h postoperatively would positively correlate with the severity of postoperative pain. The scatter plot in Figure 7 depicts the frequency of analgesics administered within 48 h following ESD. No significant differences were observed in the frequency of analgesics administered across different surgical sites within 48 h postoperatively (*H* = 3.817, *p* = 0.576, Figure 8A). However, the Kruskal-Wallis test analysis indicated that the frequency of postoperative analgesic use significantly differed among patients undergoing different surgical procedures (*H* = 12.334, *p* = 0.015, Figure 8B). Further pairwise comparisons revealed that patients who underwent ESE had a lower frequency of analgesic administration than those who underwent EFR (*p* = 0.038, Figure 8B). Additionally, Spearman correlation analysis demonstrated a negative correlation between the timing of the first analgesic administration and the frequency of postoperative analgesic use (r = −0.103, *p* = 0.013).

## Discussion

ESD is a minimally invasive endoscopic technique characterized by its high technical demands and broad clinical application. Despite its advantages, the procedure is associated with inherent risks of complications. Postoperative complications such as haemorrhage, perforation, stenosis, and infection are often the primary focus of endoscopists in the immediate post-ESD period [20–22]. However, the incidence of these major complications is relatively low and is expected to decline with ongoing advancements in endoscopic technologies. Conversely, postoperative discomfort related to APP, PONV, fever, or other minor complications is more frequently encountered but often underestimated due to its self-limiting nature. Despite being perceived as minor, these symptoms can significantly impact patients’ physical and psychological well-being, leading to lower satisfaction rates.

Moreover, given the organ-preserving nature of ESD, repeat procedures are often required, further amplifying the importance of postoperative pain management. In the present study, 570 out of 2,162 patients (26.36%) required analgesics for post-ESD pain, which is lower than the incidence reported in previous studies [8]. This finding highlights the necessity of effective pain management strategies to improve patient satisfaction and adherence to follow-up care.

Given the retrospective nature of this study and the challenges in obtaining precise postoperative pain scores, the assessment of postoperative pain was primarily based on the administration of analgesic medications. Although this method may not provide a direct measure of pain intensity, it could offer a more accurate reflection of subjective pain experiences in clinical practice due to individual variations in pain perception and tolerance. Pain is inherently subjective, with considerable inter-individual differences in its perception and threshold. Additionally, patients’ misconceptions regarding postoperative pain and the potential underestimation of pain severity by clinicians may result in a discrepancy between the number of patients requiring analgesics for moderate-to-severe pain and those who ultimately receive them [23]. This discrepancy may partly explain the lower incidence of reported postoperative pain observed in this study compared to previous reports. Implementing real-time, patient-centered pain assessment tools could enhance the accuracy of pain evaluation and optimize postoperative pain management [24].

Our findings indicated that the majority of patients undergoing ESD experienced postoperative pain predominantly during the early postoperative period, particularly within the first 12 h following surgery. This discomfort gradually subsided over time, with most patients achieving satisfactory pain relief after a single dose of postoperative analgesic medication. In our analysis, more than 40% of patients received their initial analgesic therapy within 2 h post-surgery. Additionally, Spearman correlation analysis demonstrated a negative association between the timing of initial analgesic administration and the frequency of subsequent postoperative analgesic use, suggesting that patients who received analgesics earlier were more likely to experience greater pain severity.

The COX regression analysis demonstrated that the intraoperative administration of ketorolac significantly reduced the likelihood of requiring analgesics within the first 2 h postoperatively, underscoring the importance of implementing multimodal analgesia during surgery to optimize pain management outcomes. Furthermore, preventive analgesia was found to be more effective than rescue analgesia in mitigating postoperative pain. Interestingly, our study identified that the intraoperative administration of sufentanil may paradoxically exacerbate postoperative pain following oesophageal ESD. This phenomenon could be attributed to several factors. Given the minimally invasive and short-duration nature of ESD, postoperative pain has not traditionally been a primary concern for anaesthesiologists. Consequently, standard practice typically involves the administration of 3–4 µg/kg fentanyl as part of routine intraoperative analgesia. However, in patients already at high risk of postoperative pain, the administration of sufentanil based on clinical judgement may be insufficient to fully alleviate pain during surgery, thereby contributing to the observed postoperative pain burden.

A comprehensive understanding of the risk factors influencing post-ESD pain, including its duration and intensity, is essential for anaesthesiologists to optimize perioperative pain management. Several studies have investigated the risk factors associated with postoperative pain following ESD procedures [6–10]. Consistent with previous studies [6,25], this study identified maximum specimen diameter, muscular injury, history of prior surgery, and postoperative fever as significant predictors of post-ESD pain. In addition, it was observed that smoking status (current and former), alcohol consumption, preoperative pain history, medical history of hypertension or diabetes, depth of invasion, surgical sites, types of surgery, and PONV also significantly influenced postoperative pain outcomes.

Moreover, previous studies have reported that female gender and longer surgery duration are independent risk factors [8,9]. The discrepancies in identified risk factors across studies may stem from differences in study populations and surgical methods. Most prior studies focused on single-site procedures, such as oesophageal, gastric, or colorectal ESD, whereas our study encompassed a broader cohort, including patients who underwent ESD or its derivative techniques at various anatomical sites within the digestive tract. To further elucidate site-specific risk factors, this study performed separate subgroup analyses for oesophageal, gastric, and colorectal endoscopic surgeries (see Supplementary Table B).

Furthermore, variations in the assessment intervals for postoperative pain may also contribute to discrepancies in study outcomes. This study primarily focused on identifying risk factors within the first 48 h following surgery, with findings indicating that the majority of patients experienced pain predominantly during the early postoperative period, particularly within the initial 12 h. In contrast, Zhao et al.’s study primarily examined risk factors during the 24–48 h postoperative window [6], which may partially explain the differences in reported results.

The occurrence of postoperative pain following ESD is influenced by multiple factors, with the technical proficiency of endoscopists serving as a critical determinant. Skilled endoscopists are more likely to perform procedures with greater precision, resulting in reduced tissue trauma and shorter operative durations, both of which may mitigate the incidence of postoperative pain. However, most studies investigating the risk factors associated with post-ESD pain, including the present study, have not incorporated the endoscopist’s experience as a potential risk variable. To date, only one study has reported that there was no statistically significant difference in the technical proficiency of endoscopists between patients who developed postoperative electrocoagulation syndrome (PECS) and those who did not [26]. Given the progressive improvement of endoscopist’s technical skills over time, further investigations are warranted to comprehensively evaluate the impact of operator expertise on postoperative pain outcomes.

Diverse physiological and anatomical characteristics, varying operative complexities, lesion sizes, and the depth of infiltration may contribute to distinct factors influencing postoperative pain across different anatomical sites. In the present study, patients who underwent oesophageal and duodenal surgical procedures exhibited a higher susceptibility to postoperative pain compared to those with lesions in other regions of the gastrointestinal tract. These findings are consistent with the pre-experimental study conducted by Luo et al. which demonstrated that patients undergoing oesophageal ESD experienced significantly more intense pain than those who underwent gastric ESD [12]. This phenomenon could be attributed to the absence of a serosal membrane in the oesophagus, making artificial ulcers more vulnerable to the infiltration of inflammatory agents into the muscularis propria. Additionally, the complex interplay of risk factors contributing to pain following oesophageal surgery may further exacerbate postoperative discomfort.

In the case of duodenal ESD, the narrow, tortuous, and deep anatomical location of the duodenum poses considerable technical challenges, requiring endoscopists to extend the endoscope device to maintain a clear visual field, which may lead to mechanical irritation of the intestinal wall and consequent discomfort [27]. Endoscopists frequently need to extend the endoscope device to maintain an adequate visual field, potentially resulting in mechanical irritation of the intestinal wall and subsequent discomfort [28,29]. Moreover, postoperative exposure of the surgical wound to bile and pancreatic juice may further exacerbate pain symptoms [30]. However, among patients who received postoperative analgesics, no significant differences were observed in the timing of initial analgesic administration or the frequency of analgesic use across various segments of the oesophagus and different gastrointestinal ESD procedures. The similarity in the underlying pathophysiological mechanisms of postoperative pain across different surgical sites may explain the consistent pain intensity once symptoms manifest. Nevertheless, further investigations are warranted to elucidate the precise mechanisms contributing to ESD-induced postoperative pain.

Additionally, our findings suggest that oesophageal STER holds the potential to effectively alleviate postoperative pain in patients. This effect may be attributed to the unique procedural advantage of STER, which facilitates lesion resection while preserving the integrity of the mucosal layer [31]. The intact mucosal layer serves as a protective barrier, minimizing gas and liquid leakage and thereby reducing the likelihood of microperforation and secondary inflammatory infections, both of which are significant contributors to postoperative pain [32]. However, the COX regression analysis demonstrated a significantly higher likelihood of early analgesic administration within the first 2 h postoperatively in patients who underwent STER surgery. This paradoxical finding may be explained by the prolonged operative duration of the STER procedure coupled with the progressive reduction in the efficacy of intraoperative analgesic drugs, thereby advancing the early onset of postoperative pain [32,33].

The complexity of the surgical procedure serves as a significant risk factor for postoperative pain. Although no significant differences were observed in the time to first analgesic administration among patients who underwent different types of surgery, our findings revealed that patients who underwent EFR surgery required postoperative analgesics more frequently than those who underwent ESE surgery. This discrepancy could be attributed to the invasive nature of the EFR procedure, which involves resection extending into the muscular layer or even the serosa, potentially causing greater damage to nerve endings and blood vessels. Such extensive tissue trauma may elicit a more intense inflammatory response and exacerbate visceral pain.

Additionally, the active perforation intentionally induced during EFR surgery results in pneumoperitoneum, while the application of titanium clips to close the defect may exert traction on surrounding tissues, potentially triggering postoperative referred pain. Even when intraoperative closure is deemed successful, local peritonitis may still occur due to the leakage of gastric acid or bacteria, leading to persistent abdominal pain [34]. Currently, limited literature is available regarding postoperative pain following EFR surgery, underscoring the need for further clinical investigations to gain deeper insights into its underlying mechanisms and optimize pain management strategies.

In summary, the pathogenesis of postoperative pain following ESD is multifactorial and cannot be attributed to a single influencing factor. A complex interplay of intrinsic patient characteristics, surgical techniques, and analgesic administration strategies collectively contributes to the incidence and severity of postoperative pain. Therefore, a comprehensive, multidisciplinary approach is essential to optimize pain management. Endoscopists should prioritise refining their technical skills to minimize the duration of the procedure, reduce intestinal cavity inflation, and mitigate electrocoagulation-induced injury. Simultaneously, anaesthesiologists should perform individualized preoperative risk assessments to identify patients at high risk for postoperative pain and implement pre-emptive or multimodal analgesic protocols to improve postoperative outcomes.

Our study represents an initial investigation into the severity of postoperative pain and its associated risk factors across various segments of the gastrointestinal tract following minimally invasive digestive endoscopic procedures. Additionally, a comparative analysis was conducted to evaluate the timing and frequency of initial analgesic administration across different anatomical sites and surgical techniques. However, several potential limitations should be acknowledged. Firstly, this study was conducted at a single centre, which may limit the generalizability of the findings to broader populations. Variations in surgical techniques, analgesic protocols, and patient demographics across institutions could significantly impact postoperative pain outcomes. Future multicentre prospective studies are essential to validate our findings and enhance the robustness of the conclusions. Our centre also plans to further explore whether prophylactic analgesia with oxycodone can effectively alleviate post-ESD pain. Secondly, several potentially confounding variables were not evaluated, including histological subtype, presence of *H. pylori* infection, types of submucosal injection solutions, qualifications of the operators, types of endoscopic knives used, and postoperative administration of PPIs or antibiotics. These factors may independently influence postoperative pain outcomes and should be systematically investigated in future studies to provide a more comprehensive understanding of the underlying mechanisms.

## Conclusions

In conclusion, our study demonstrated that a considerable proportion of patients undergoing ESD and its derivative techniques required one or more doses of analgesic medication within 48 h post-procedure, with the associated risk factors being multifaceted. Patients who underwent oesophageal and duodenal ESD or EFR of the stomach exhibited a higher likelihood of requiring postoperative analgesics. Notably, the intraoperative administration of ketorolac significantly delayed the time to first analgesic administration and reduced the frequency of analgesic use, underscoring the potential benefits of pre-emptive multimodal analgesia. These findings highlight the necessity for both endoscopists and anaesthesiologists to maintain a heightened awareness of postoperative pain following ESD, particularly in high-risk patients. Implementing pre-emptive or aggressive pain management strategies may improve postoperative comfort and overall patient satisfaction. However, as this study was a retrospective single-centre analysis, the generalizability of the findings remains limited. Future prospective RCTs across multiple centres are warranted to further validate these observations and optimize postoperative pain management protocols.

## Supplementary Material

SuppTable B.docx

SuppTable A.docx

## Data Availability

Reasonable requests for access to the datasets used and/or analyzed during this study can be made to the corresponding.
