# Supplementary material for: Painkiller administration after endoscopic submucosal dissection surgery: a retrospective real-world study
Source: Ann Med. 2025 May 10;57(1):2499698. doi: 10.1080/07853890.2025.2499698 (PMC12068328; doi:10.1080/07853890.2025.2499698)
Supplement: SuppTable B.docx [file IANN_A_2499698_SM0957.docx]

Table B Multivariate analysis of risk factors affecting postoperative pain following esophageal ESD, gastric ESD and colorectal ESD.

| Variables | Multivariate analysis | | | | | |
| --- | --- | --- | --- | --- | --- | --- |
|  | Esophagus | | Stomach | | Colorectum | |
|  | OR (95%CI) | *P* | OR (95%CI) | *P* | OR (95%CI) | *P* |
| Age (y) |  |  |  |  |  |  |
| (18-39) vs. (40-49) | 0.65 (0.14-3.02) | 0.583 |  |  |  |  |
| (18-39) vs.(50-59) | 0.21 (0.05-0.87) | 0.031 |  |  |  |  |
| (18-39) vs.(60-69） | 0.24 (0.06-0.97) | 0.044 |  |  |  |  |
| (18-39) vs. (70-79) | 0.23 (0.05-0.95) | 0.043 |  |  |  |  |
| (18-39) vs. (≥80) | 0.14 (0.03-0.80) | 0.027 |  |  |  |  |
| Gender (female vs. male) | 0.13 (0.02-0.77) | 0.024 |  |  |  |  |
| Smoking |  |  |  |  |  |  |
| non-smokers vs. present smokers | 1.77 (1.06-2.97) | 0.030 |  |  |  |  |
| non-smokers vs. Former smokers | 3.91 (1.67-9.14) | 0.002 |  |  |  |  |
| Drinking |  |  |  |  |  |  |
| non-drinkers vs. present drinkers | 0.53 (0.31-0.89) | 0.016 |  |  | 0.13 (0.02-0.70) | 0.018 |
| non-drinkers vs. former drinkers | 0.60 (0.22-1.59) | 0.300 |  |  | 0.10 (0.01-1.61) | 0.105 |
| History.of.surgery (no vs. yes) | 1.83 (1.29-2.58) | <0.001 | 2.57 (1.62-4.08) | <0.001 |  |  |
| Hypertension (no vs. yes) | 1.68 (1.09-2.59) | 0.019 |  |  |  |  |
| Diabetes (no vs. yes) | 5.38 (3.21-9.04) | <0.001 | 5.23 (2.81-9.72) | <0.001 |  |  |
| Preoperative pain history (no vs. yes) | 2.84 (2.03-3.97) | <0.001 | 1.87 (1.18-2.96) | 0.007 |  |  |
| Operation |  |  |  |  |  |  |
| ESD vs. ESE | 1.37 (0.32-5.80) | 0.668 |  |  |  |  |
| ESD vs. EFR | NA |  |  |  |  |  |
| ESD vs. STER | 0.42 (0.19-0.96) | 0.039 |  |  |  |  |
| ESD vs. Combined operation | 3.31 (0.26-42.89) | 0.360 |  |  |  |  |
| Maximum specimen diameter (cm) | 1.11 (1.01-1.21) | 0.028 | 1.25 (1.06-1.48) | 0.007 |  |  |
| Depth of infiltration |  |  |  |  |  |  |
| Mucous layer vs. Submucosa |  |  | 4.41 (2.12-9.13) | <0.001 |  |  |
| Mucous layer vs. Lamina musculi propria |  |  | 1.79 (0.70-4.56) | 0.223 |  |  |
| Muscular injury (no vs. yes) |  |  |  |  | 3.60 (1.10-11.76) | 0.034 |
| Surgery time (min) | 1.01 (1.00-1.02) | 0.011 |  |  | 1.12 (1.01-1.25) | 0.039 |
| Duration of anesthesia (min) |  |  | 1.04 (1.00-1.09) | 0.044 |  |  |
| Sufentanil (no vs. yes) | 9.60 (3.92-23.48) | <0.001 |  |  |  |  |
| PONV (no vs. yes) | 3.63 (2.44-5.41) | <0.001 | 2.32 (1.40-3.85) | 0.001 |  |  |
| Postoperative fever (no vs. yes) |  |  | 2.29 (1.25-4.21) | 0.007 |  |  |

ESD, Endoscopic submucosal dissection. ESE, Endoscopic submucosal excavation. EFR, Endoscopic full-thickness resection. STER, Submucosal tunnel endoscopic resection. PONV, Postoperative nausea and vomiting.
