# Supplementary material for: Painkiller administration after endoscopic submucosal dissection surgery: a retrospective real-world study
Source: Ann Med. 2025 May 10;57(1):2499698. doi: 10.1080/07853890.2025.2499698 (PMC12068328; doi:10.1080/07853890.2025.2499698)
Supplement: SuppTable A.docx [file IANN_A_2499698_SM0956.docx]

Table A Univariate and multivariate analysis of risk factors for post-ESD pain with raw data.

| Variables | Univariate analysis | | Multivariate analysis | |
| --- | --- | --- | --- | --- |
|  | Odds ratio  (95%CI) | *P* value | Odds ratio (95%CI) | *P* value |
| Age (y) |  |  |  |  |
| (18-39) *vs.* (40-49) | 0.77 (0.40-1.48) | 0.430 |  |  |
| (18-39) *vs.*(50-59) | 1.06 (0.60-1.88,) | 0.843 |  |  |
| (18-39) *vs.*(60-69） | 1.01 (0.57-1.78) | 0.975 |  |  |
| (18-39) *vs.* (70-79) | 1.40 (0.78-2.51) | 0.261 |  |  |
| (18-39) *vs.* (≥80) | 1.03 (0.42-2.52) | 0..949 |  |  |
| Gender (female *vs.* male) | 0.82 (0.67-1.01) | 0.060 |  |  |
| Smoking |  |  |  |  |
| non-smokers *vs*. present smokers | 0.92 (0.73-1.16) | 0..487 | 1.80 (1.20-2.71) | 0.004 |
| non-smokers *vs*. Former smokers | 2.35 (1.63-3.40) | < 0.001 | 3.55 (1.86-6.78) | < 0.001 |
| Drinking |  |  |  |  |
| non-drinkers *vs*. present drinkers | 0.75 (0.60-0.94) | 0..013 | 0.43 (0.29-0.65,) | < 0.001 |
| non-drinkers *vs*.former drinkers | 1.52 (0.97-2.38) | 0.071 | 0.41 (0.19-0.87) | 0.020 |
| ASA |  |  |  |  |
| I *vs.* II | 3.57 (0.83-15.32) | 0..087 |  |  |
| I *vs.* III | 0.00 (0.00-Inf) | 0.966 |  |  |
| History of surgery (no *vs.* yes) | 1.76 (1.43-2.18) | < 0.001 | 1.85 (1.44-2.39) | < 0.001 |
| Hypertension (no *vs.* yes) | 2.79 (2.23-3.48) | < 0.001 | 1.66 (1.23-2.24) | < 0.001 |
| Diabetes (no *vs.* yes) | 6.61 (5.01-8.72) | < 0.001 | 5.11 (3.59-7.28) | < 0.001 |
| Preoperative pain history (no *vs.* yes) | 2.29 (1.86-2.82) | < 0.001 | 2.32 (1.81-2.97) | < 0.001 |
| Surgical site |  |  |  |  |
| Esophagus *vs.* EGJ | 0.30 (0.19-0.46) | < 0.001 | 0.21 (0.13-0.34) | < 0.001 |
| Esophagus *vs.* Stomach | 0.38 (0.30-0.49,) | < 0.001 | 0.26 (0.18-0.38) | < 0.001 |
| Esophagus *vs.* Duodenum | 0.96 (0.32-2.83) | 0.937 | 0.77 (0.19-3.02) | 0.703 |
| Esophagus *vs.* Colorectum | 0.25 (0.16-0.40) | < 0.001 | 0.20 (0.10-0.40) | < 0.001 |
| Esophagus *vs.* Multisite  Operation | 1.00 (0.60-1.65) | 0.992 | 0.47 (0.23-0.97) | 0.042 |
| ESD *vs*. ESE | 0.72 (0.50-1.03) | 0.074 | 0.71 (0.39-1.28) | 0.256 |
| ESD *vs*. EFR | 1.68 (0.99-2.85) | 0.056 | 1.58 (0.70-3.59) | 0.272 |
| ESD *vs*. STER | 1.21 (0.76-1.91) | 0..422 | 0.41 (0.19-0.87) | 0.021 |
| ESD *vs*. Combined operation | 2.66 (1.38-5.12) | 0.004 | 1.33 (0.51-3.49) | 0.558 |
| Maximum specimen diameter (cm) | 1.20 (1.14-1.27) | < 0.001 | 1.14 (1.06-1.22) | < 0.001 |
| Depth of infiltration |  |  |  |  |
| Mucous layer *vs*. Submucosa | 1.82 (1.20-2.77) | 0.005 | 4.40 (2.54-7.61) | < 0.001 |
| Mucous layer *vs*. Lamina muscularis propria | 1.11 (0.86-1.44) | 0.421 | 2.01 (1.11-3.64) | 0.021 |
| Muscular injury (no *vs.* yes) | 1.29 (1.04-1.60) | 0.019 | 1.81 (1.27-2.60) | 0.001 |
| Surgery time (min) | 1.01 (1.01-1.01) | < 0.001 | 1.00 (0.99-1.01) | 0.539 |
| Duration of anesthesia (min) | 1.01 (1.01-1.01) | < 0.001 | 1.01 (1.00-1.01) | 0.167 |
| Fentanyl (no vs. yes) | 1.97 (1.20-3.24) | 0.008 | 0.80 (0.38-1.70) | 0.564 |
| Sufentanil (no vs. yes) | 1.40 (1.00-1.95) | 0.050 |  |  |
| Oxycodone (no vs. yes) | 0.00 (0.00-Inf) | 0.972 |  |  |
| Nalbuphine (no vs. yes) | 0.92 (0.37-2.31) | 0.862 |  |  |
| Ketorolac (no vs. yes) | 1.75 (1.11-2.74) | 0.015 | 1.38 (0.80-2.38) | 0.252 |
| PONV (no *vs.* yes) | 2.65 (2.10-3.35) | < 0.001 | 2.93 (2.20-3.89) | < 0.001 |
| Postoperative fever (no *vs.* yes) | 1.85 (1.40-2.45) | < 0.001 | 1.52 (1.08-2.15) | 0.018 |

EGJ, Esophagogastric junction. ESD, Endoscopic submucosal dissection. ESE, Endoscopic submucosal excavation. EFR, Endoscopic full-thickness resection. STER, Submucosal tunnel endoscopic resection. PONV, Postoperative nausea and vomiting.
